# The Vaccine Book

**DOI:** 10.3201/eid1007.030910

**Published:** 2004-07

**Authors:** Bruce G. Weniger

**Affiliations:** * Centers for Disease Control and Prevention, Atlanta, GA, USA

**Keywords:** book review, vaccines, vaccination, immunization, public health, economics, epidemiology

Few fields in medical science involve as wide a range of specialties and expertise as vaccinology. It encompasses the research, development, and manufacturing processes of vaccines, their incorporation into immunization programs, and the logistic and clinical aspects of their use. Commissioning experts to write chapters with a minimum of jargon, minutiae, and redundancy for a book with a target audience of immunologists, microbiologists, clinical trial specialists, epidemiologists, economists, policy-setting public health officials, and practitioners who administer the resulting products and provide follow-up care, is challenging. But the experienced editors of this book have achieved this goal. Dr. Bloom was previously a *Mycobacterium* immunologist at the Albert Einstein School of Medicine and is now dean of the Harvard School of Public Health. Dr. Lambert is a vaccine immunologist at the University of Geneva.

The Vaccine Book first covers the impact of disease, including chapters on vaccine economics and finance policy, and the potential for widespread vaccination to change the epidemiology of the target disease. One example is the herd effect of childhood rubella vaccination, which postpones infection in nonimmunized women into their childbearing years. The next section reviews the immune system, and here lies the book's greatest disappointment. Its chapter on basic immunology is confusing and presumes familiarity with terms and concepts without antecedent explanation. It lacks a logical flow in describing what is yet known of the (infinitely?) complex immune system and its many "up-" and "down-regulating" feedback loops. Readers hoping for a chapter-length "Immunology 101" course would be advised to turn elsewhere (*1**,**2*)

The phased stages of clinical trials are covered in excellent chapters by accomplished authors with practical insights. Another section shows how knowledge of microbial pathogenesis can affect vaccine design, including Rolf Zinkernagel's well-written chapter on immunologic memory. Another chapter on parasite pathogenesis, however, delves too deeply into the immunity of *Leishmania* as a case study.

Stanley Plotkin's thoughtful overview of the 11 disease-specific chapters annotates new vaccine technologies as well as current issues of debate, such as replacing the live oral polio vaccine worldwide with injectable, inactivated polio vaccine once the eradication program breaks the chain of wild-virus circulation, to avoid reverse mutations and resulting vaccine-associated paralysis. Plotkin also provides a comprehensive table of vaccine types currently available or in active clinical development.

Remaining sections of The Vaccine Book cover the ethics of research and use of vaccines, their safety and controversies, and their introduction into healthcare systems. The editors conclude with major future challenges, such as circumventing microbial escape, vaccines for chronic and autoimmune diseases, and maintaining public support of immunization in the face of anti-vaccine movements.

The breadth of vaccinology inevitably requires leaving out some topics. There is no chapter on measles vaccines, used universally for a major cause of childhood death and disability. Manufacturing steps such as fermentation, purification, formulation, fill, and finish are not described. There is little on quality assurance and regulation, such as the investigational new drug application process and current good manufacturing practice, although good clinical practice is mentioned. Despite these gaps, compared to this field's authoritative encyclopedia (*3*), at three times The Vaccine Book's mass and four times its pages, this handy 1.1-kg compilation is a more comfortable read, indeed.
